# Effects of antipsychotic and anticholinergic medications on cognition in chronic patients with schizophrenia

**DOI:** 10.1186/s12888-023-04552-y

**Published:** 2023-01-24

**Authors:** Chadia Haddad, Pascale Salameh, Hala Sacre, Jean-Pierre Clément, Benjamin Calvet

**Affiliations:** 1 Inserm U1094, IRD UMR270, Univ. Limoges, CHU Limoges, EpiMaCT - Epidemiology of Chronic Diseases in Tropical Zone, Institute of Epidemiology and Tropical Neurology, OmegaHealth, Limoges, France; 2 Centre Mémoire de Ressources Et de Recherche du Limousin, Centre Hospitalier Esquirol, 87000 Limoges, France; 3 Research Department, Psychiatric Hospital of the Cross, P.O. Box 60096, Jal Eddib, Lebanon; 4 INSPECT-LB (Institut National de Santé Publique, d’Épidémiologie Clinique et de Toxicologie-Liban), Beirut, Lebanon; 5 School of Health Sciences, Modern University for Business and Science, Beirut, Lebanon; 6 School of Medicine, Lebanese American University, Byblos, Lebanon; 7 Department of Primary Care and Population Health, University of Nicosia Medical School, 2417 Nicosia, Cyprus; 8 Faculty of Pharmacy, Lebanese University, Hadat, Lebanon; 9 Pôle Universitaire de Psychiatrie de L’Adulte, de l’Agée Et d’Addictologie, Centre Hospitalier Esquirol, 87000 Limoges, France

**Keywords:** Schizophrenia, Anticholinergic activity, Antipsychotics, Cognition

## Abstract

**Background:**

Patients with psychosis frequently use a variety of psychotropic medicines, many of which have anticholinergic effects that can impair cognition. Therefore, this study aimed to evaluate whether there is an association between medications used for neuropsychological disorders/symptoms and cognition in patients with schizophrenia, focusing on their anticholinergic load and antipsychotic doses.

**Study design:**

A cross-sectional study between July 2019 and Mars 2020 at the Psychiatric Hospital of the Cross-Lebanon enrolled 120 inpatients diagnosed with schizophrenia. The total anticholinergic burden was calculated based on the Anticholinergic Drug Scale (ADS), and the chlorpromazine equivalent dose was calculated using the Andreasen method to assess the relative antipsychotic dose. Also, the objective cognition was assessed using the Brief Assessment of Cognition in Schizophrenia (BACS) tool.

Study results.

A significantly higher BACS total score (*r* = -0.33, *p* < 0.001), higher verbal memory (*r* = -0.26, *p* = 0.004), higher working memory (*r* = -0.20, *p* = 0.03), higher motor speed (*r* = -0.36, *p* < 0.001), and higher attention and speed of information processing (*r* = -0.27, *p* = 0.003) were significantly associated with lower chlorpromazine equivalent dose. Higher ADS (Standardized Beta (SB) = -.22; *p* = .028), higher chlorpromazine equivalent dose (SB = -.30; *p* = .001), and taking mood stabilizer medications (SB = -.24; *p* = .004) were significantly associated with lower cognition.

**Conclusion:**

This study confirms that the cognitive functions of chronic patients with schizophrenia may be affected by medications and their anticholinergic burden. More studies are needed to explain the role of cholinergic neurotransmission and general neurochemical mechanisms in the cognitive impairment of patients with schizophrenia.

**Supplementary Information:**

The online version contains supplementary material available at 10.1186/s12888-023-04552-y.

## Introduction

Cognitive impairment occurs in most people with schizophrenia and has been suggested as a core feature of the illness [1, 2]. It presents before the onset of psychosis, stays reasonably stable throughout the disease, and is highly linked to functional prognosis [3, 4]. Most patients with schizophrenia have a generalized cognitive impairment, and some present impairment in several specific areas of functioning [5], such as speed of processing, attention, working memory, verbal learning and memory, visual learning and memory, reasoning and problem-solving, and social cognition [6, 7]. As poor performance on neurocognitive measures is more directly connected to functional outcomes than symptoms [8, 9], improving cognitive functioning is considered an essential component of schizophrenia treatment and quality of life [10].

Treatment of cognitive impairments has become a focus of research due to their negative influence on functional outcomes. Given that antipsychotic (AP) medications are the cornerstone of schizophrenia treatment, the link between APs and cognition remains controversial despite a large number of studies examining this relationship. Meta-analyses have found that the use of both first (FGA) and second (SGA) generation antipsychotics medications in schizophrenia has been linked to slight to moderate cognitive improvement [11–13]. Historically, the first generation of antipsychotics was thought to have little effect on cognitive performance or even be harmful [14]. The development of SGA sparked hopes that these newer medications would improve cognitive function over the first generation [14]. However, the Clinical Antipsychotic Trials of Intervention Effectiveness (CATIE) study, which included a large sample of chronic patients with schizophrenia, found that APs are highly similar in their activities across chemical classes [15]. This similarity extends to the effects APs have on cognition [15]. Despite their beneficial effects on cognition, many APs may have negative impacts. First-generation APs have been found to impair procedural learning and memory, particularly at high doses [13, 16]. High doses of mono or polypharmacy have also been linked to severe cognitive impairment [17, 18]. Furthermore, it has been demonstrated that numerous cognitive domains, including memory, visuospatial, language, attention, and delayed memory, improve considerably when AP doses are lowered [19, 20]. Conversely, in a meta-analysis of older patients with schizophrenia, medication status or chlorpromazine equivalent dosage failed to show a meaningful relationship with cognition across 1–6 years of follow-up [21].

Medications with a high anticholinergic activity can have a detrimental effect on cognition [22]. A recent systematic literature review of 17 articles has demonstrated that medication with increased anticholinergic load negatively affected the neurocognitive performance of patients with schizophrenia, as shown in most studies [23]. Patients with psychosis frequently use several psychotropic medicines, many of which have anticholinergic effects to various extents [24]. Also, several non-psychotropic drugs used by patients with schizophrenia, such as mood stabilizers, antidepressants, and anxiolytics, have anticholinergic effects [23, 25–28], and many additional anticholinergic medications are frequently co-prescribed to alleviate the extrapyramidal side effects caused by antipsychotics, particularly typical agents [29]. Most studies on the relationship between anticholinergic medications and cognition have been conducted among the elderly, where the anticholinergic load is associated with increased delirium, falls, and cognitive impairments [22, 30, 31]. A review on the anticholinergic burden and cognition in the elderly found a link between anticholinergic medication use and poorer cognition, including specific deficits in processing speed, attention, language, problem-solving, and psychomotor performance [22]. Similarly, anticholinergic load and length of anticholinergic medication use were related to the deterioration in cognition in an 8-year longitudinal study among patients with Parkinson’s disease [32]. A few studies have examined the relationship between the anticholinergic burden and cognition in schizophrenia. Recent research among 1,120 patients with schizophrenia found that a higher anticholinergic burden was associated with worse cognitive performance [33]. A study among 705 Chinese patients with schizophrenia aged 21 to 55 found that those with a higher medication anticholinergic burden performed worse in cognitive tasks, particularly executive functioning, memory/fluency, and processing speed [34]. Another study among patients with psychotic disorders (206 schizophrenia, 131 schizoaffective, and 146 psychotic bipolar disorder) found that, among schizophrenia participants, the anticholinergic drug burden score was significantly associated with lower performance on cognitive tasks, especially verbal memory, token motor, and symbol coding tests [35]. It also revealed that higher antipsychotic doses were linked to poorer token motor performance across all diagnostic categories [35]. A study among 60 older patients with schizophrenia found that the anticholinergic burden was associated with worse spatial working, immediate memory, and visuospatial ability but not with attention, executive function, language, or reaction time [36].

This study hypothesized that higher anticholinergic load and higher antipsychotic doses are linked to worse cognitive function in patients with schizophrenia. Thus, a better understanding of the adverse cognitive consequences of anticholinergic burden and antipsychotic dose might guide clinicians in prescribing treatment. Therefore, this study aimed to evaluate whether there is an association between the medications used for neuropsychological disorders/symptoms and cognition in patients with schizophrenia, focusing on their anticholinergic load and antipsychotic doses.

## Methods

### Study design and participants

A cross-sectional study between July 2019 and Mars 2020 at the Psychiatric Hospital of the Cross-Lebanon enrolled 120 inpatients diagnosed with schizophrenia. Patients included were between 18 and 60, had an education level of more than five schooling years, met the Diagnostic and Statistical Manual of Mental Disorders Fifth Edition (DSM-5) criteria for schizophrenia, were in the remission phase, received antipsychotic medication, and were clinically stable. Exclusion criteria were brain trauma, neurological problem, or current substance use disorder that would influence cognitive performance. This study is a part of a large project, and the same method was used in a previous study [37].

### Medication assessment

Medication data were retrieved from the medical records of participants. It included mainly antipsychotics, mood stabilizers, benzodiazepines, anticholinergics, and antidepressants.

Mood stabilizers comprised lithium carbonate and antiepileptic medications (valproic acid, carbamazepine, pregabalin, topiramate, and phenytoin). The benzodiazepines included lorazepam, clonazepam, alprazolam, diazepam, alprazolam, and bromazepam. Anticholinergics consisted of trihexyphenidyl. Antidepressants were divided into two groups, i.e., tricyclic antidepressants (TCA, including amitriptyline, clomipramine, and imipramine) and other antidepressants (venlafaxine, sertraline).

The total anticholinergic burden was calculated based on the updated version of the Anticholinergic Drug Scale (ADS), where each medication was assigned a numerical value from 0 to 3, depending on their anticholinergic strength, and the overall ADS score for a patient was calculated by summing the values of all scheduled medications used by each participant [28]. This scale is the most comprehensive currently available for quantifying the anticholinergic burden for most medicines used to treat psychotic symptoms [28].

Serum anticholinergic activity (SAA) is considered the current gold standard in quantifying anticholinergic burden [28]. However, it is only quantified in a small number of research laboratories. An ADS scale might help determine who is the most at risk for adverse side effects and offer guidance in interventions [28]. Previous studies have shown that the ADS score was significantly associated with SAA, suggesting it is a helpful tool for assessing anticholinergic burden [28, 38]. However, using ADS ratings to classify medications is a method of limited accuracy since there will be variations in anticholinergic potencies across medications within each group, regardless of the accuracy of the classification. Also, SAA might be influenced by endogenous substances, which are not measurable by the ADS scale, which only evaluates anticholinergic characteristics of medications.

Furthermore, antipsychotic medications were divided into second-generation antipsychotics or SGA (risperidone, clozapine, olanzapine, quetiapine, and paliperidone) and first-generation antipsychotics or FGA (haloperidol, chlorpromazine, pimozide, zuclopenthixol, fluphenazine, and perphenazine). The sum of antipsychotics taken was calculated for each patient. The chlorpromazine equivalent dose was calculated using the Andreasen method to assess the relative antipsychotic dose [39], and the doses of benzodiazepines were calculated using the equivalent benzodiazepine calculator based on the valium equivalence [40].

### Neuropsychological test measures

All participants were assessed by the Brief Assessment of Cognition in Schizophrenia (BACS) cognitive battery [41], recently validated in Lebanon [37]. The BACS includes six subscales: list learning (verbal memory), digit sequencing (working memory), token motor task (psychomotor function), semantic fluency (verbal fluency), symbol coding (attention and speed of information processing), and Tower of London (executive function).

### The positive and negative syndrome scale (PANSS)

The PANSS, validated in Arabic [42], is a 30-item questionnaire organized into three subscales: positive symptoms (7 items), negative symptoms (7 items), and general psychopathology (16 items) [43]. All items are scored from 1 (absence of symptoms) to 7 (extremely severe symptoms) [43]. The total score was calculated by summing all answers, with higher scores indicating more severe symptoms [43].

### Statistical analysis

Data were analyzed using SPSS software version 25. Categorical data were reported as absolute frequencies and percentages, whereas quantitative variables were expressed as means and standard deviations. The BACS composite score (z-score) was calculated by standardizing the total score over that of the healthy control group. This calculation was based on a healthy control group from a previously validated study using the same sample of patients with schizophrenia [37]. The independent-sample t-test was used to compare the composite score of the BACS total score and subtests with the medication groups. The Pearson correlation test was used to evaluate the association between continuous variables.

To assess the association between each subtest of the BACS scale with the ADS scale, the latter was grouped into five categories based on their ADS scores: no anticholinergic burden (ADS score = 0), low anticholinergic burden (ADS score = 1 or 2), moderate anticholinergic burden (ADS score = 3 or 4), high anticholinergic burden (ADS score = 5 or 6), or very high anticholinergic burden (ADS score = above 6). The ANOVA test was used to compare these groups and the BACS subscales (ADS = 0).

A series of multivariable linear regression analyses were conducted, taking the BACS scale and subtests as the dependent variables and the neuropsychological medications, their anticholinergic burden, and chlorpromazine equivalent dose as the independent variables. The adjusted variables were symptom severity (PANSS total score), gender, education level, age, duration of illness, and depression. Significance was set at a *p* < 0.05.

## Results

### Sample characteristics

Table 1 shows the sociodemographic characteristics of patients with schizophrenia. More than half of the participants were male (59.2%), single (81.7%), with a secondary level of education (50.0%), and 35.3% had a family history of psychiatric illness. Mean illness and hospitalization lengths were 20.6 ± 9.8 and 12.4 ± 8.5 years, respectively. The mean number of hospitalizations was 6.3 ± 5.6 times, and the mean age was 48.4 ± 7.6 years.

**Table 1:** Sociodemographic characteristics of the studied sample (*N* = 120)

	**Frequency (%)**
**Male Gender**	71 (59.2%)
**Education level**	
Complementary	41 (34.2%)
Secondary	60 (50.0%)
University	19 (15.8%)
**Marital Status**	
Single/ Divorced/ Widowed	110 (91.7%)
Married	10 (8.3%)
**Monthly income**	
No income	27 (22.5%)
< 1000 $	64 (53.3%)
1000—2000 $	27 (22.5%)
> 2000 $	2 (1.7%)
**Presence of family history of psychiatric illness**	42 (35.3%)
	**Mean ± SD**
**Age in years**	48.43 ± 7.62
**Duration of hospitalization in years**	12.47 ± 8.56
**Duration of illness in years**	20.64 ± 9.79
**Number of hospitalizations**	6.32 ± 5.65
**Total PANSS scale**	82.88 ± 27.05

### Medications used

FGAs were the most used (76.7%), followed by SGAs (50.0%). Only 36.1% of the patients were treated with mood stabilizers; among those patients, 13.3% took lithium, and 48.3% took antiepileptic medications. Also, 37.5% were treated with benzodiazepines and 70.8% with anticholinergics. Considering antidepressants, 7.5% of the participants took TCAs, and 5.0% used the SSRI medication types. The mean chlorpromazine equivalent dose was 1041.6 [Min: 0.5; Max: 4502.0], the mean Benzodiazepine equivalent dose was 15.03 [Min 5.00; Max 48.00] and the mean duration of medication treatment was 54.7 ± 29.5 months. The mean ADS score was 7.18 ± 3.11, and the mean number of antipsychotics used was 1.85 ± 0.94 (Table 2).

**Table 2:** Description of the type of medications used by patients with schizophrenia

	**Frequency (%)**
**Antipscyhotic medication**	
**Second generation antipsychotics**	
Yes	60 (50.0%)
No	60 (50.0%)
**First generation antipsychotics**	
Yes	92 (76.7%)
No	28 (23.3%)
**Mood stabilizer medications *(Lithium family group and antiepileptic medications)***	
Yes	65 (36.1%)
No	115 (63.9%)
**Lithium carbonate**	
Yes	16 (13.3%)
No	104 (86.7%)
**Antiepileptics**	
Yes	58 (48.3%)
No	62 (51.7%)
**Benzodiazepines**	
Yes	45 (37.5%)
No	75 (62.5%)
**Anticholinergics**	
Yes	85 (70.8%)
No	35 (29.2%)
**Antidepressants medications**	
Yes	15 (8.3%)
No	165 (91.7%)
**Tricyclic antidepressants**	
Yes	9 (7.5%)
No	111 (92.5%)
**Other antidepressants**	
Yes	6 (5.0%)
No	114 (95.0%)
Other types of medications*	
Yes	61 (50.8%)
No	59 (49.2%)
	**Mean ± SD**
**Chlorpromazine equivalent dose**	1041.6 [Min .5; Max 4502.0]
**Benzodiazepine (Valium) equivalent dose**	15.03 [Min 5.00; Max 48.00]
**Anticholinergic Drug Scale (ADS)**	7.18 ± 3.11
**Duration of medication treatment (in months)**	54.7 ± 29.5
**Number of antipsychotics used**	1.85 ± 0.94

^*^Second generation antipsychotic: risperidone, clozapine, olanzapine, quetiapine, and paliperidone

First generation antipsychotic: haloperidol, chlorpromazine, pimozide, zuclopenthixol, fluphenazine, and perphenazine

Antiepileptics: valproic acid, carbamazepine, pregabalin, topiramate, phenytoin

Benzodiazepines: lorazepam, clonazepam, alprazolam, diazepam, alprazolam, bromazepam

Anticholinergics: trihexyphenidyl

Tricyclic antidepressants: amitriptyline, clomipramine, imipramine

other antidepressants: venlafaxine, sertraline

Other types of medications: Anticoagulants, supplements, antimuscarinics, antiarrhythmics, antiparkinsonians, antiasthmatic agents, vitamins, thyroid medications, stomach protection, antidiabetics, statins, antihypertensives, and proton-pump inhibitors

### Comparisons of medications used and cognition in patients with schizophrenia

The association between antipsychotic medications and cognition adjusted for treatment duration, illness length, and chlorpromazine equivalent dose is displayed in Table 3.

**Table 3:** Association between Antipsychotic medication and cognitive function

	**Second generation antipsychotics**		**First generation antipsychotics**	
	**No**	**Yes**	**No**	**Yes**
	**Mean ± SE**	**Mean ± SE**	**Mean ± SE**	**Mean ± SE**
**BACS (global score)**	-2.86 ± 0.15	-2.86 ± 0.14	-2.87 ± 0.25	-2.86 ± 0.12
*p-value*	0.979		0.959	
**Verbal memory (List learning)**	-2.10 ± 0.14	-2.13 ± 0.13	-2.10 ± 0.22	-2.12 ± 0.11
*p-value*	0.854		0.943	
**Working memory (Digit sequencing)**	-2.05 ± 0.17	-1.87 ± 0.16	-2.15 ± 0.27	-1.90 ± 0.13
*p-value*	0.455		0.431	
**Motor speed (Token motor task)**	-2.34 ± 0.13	-2.38 ± 0.12	-2.29 ± 0.21	-2.38 ± 0.10
*p-value*	0.819		0.724	
**Verbal fluency (Semantic, alphabetical)**	-1.43 ± 0.13	-1.59 ± 0.12	-1.58 ± 0.21	-1.50 ± 0.10
*p-value*	0.394		0.725	
**Attention and speed of information processing (Symbol coding)**	-2.44 ± 0.16	-2.38 ± 0.15	-2.36 ± 0.25	-2.43 ± 0.12
*p-value*	0.803		0.815	
**Executive function (Tower of London)**	-2.30 ± 0.29	-1.96 ± 0.27	-2.31 ± 0.47	-2.07 ± 0.22
*p-value*	0.415		0.661	

Note: the association between the antipsychotics used and the BACS total score and subtests was adjusted for treatment duration, illness length, and chlorpromazine equivalent dose

No significant association was found between all objective cognitions (BACS total score and subtests) and antipsychotic use (whether taking or not FGA and SGA medications) (*p* > 0.05 for all).

When assessing cognition in participants taking other medications, a significantly lower mean total cognitive deficit (BACS total score) was found among those taking mood stabilizers (more deficit). Also, a significantly lower mean score of working memory, motor speed, verbal fluency, attention and executive function was found among those taking mood stabilizers treatment. Also, a lower mean verbal memory was found among those taking anticholinergic medications (Table 4). There was no significant association between medication doses and cognition (Supplementary Table S1).

**Table 4:** Association between psychiatric medication (mood stabilizer, Benzodiazepine, anti-cholinergic, and anti-depressant) and cognitive function

	**Mood stabilizer**		**Benzodiazepine**		**Anticholinergics**		**TCA Anti—depressants**		**Other Anti—depressants**	
	**No**	**Yes**	**No**	**Yes**	**No**	**Yes**	**No**	**Yes**	**No**	**Yes**
	**Mean ± SE**	**Mean ± SE**	**Mean ± SE**	**Mean ± SE**	**Mean ± SE**	**Mean ± SE**	**Mean ± SE**	**Mean ± SE**	**Mean ± SE**	**Mean ± SE**
**BACS (global score)**	-2.50 ± 0.15	-3.15 ± 0.13	-2.75 ± 0.13	-3.04 ± 0.17	-2.53 ± 0.20	-2.99 ± 0.12	-2.85 ± 0.10	-2.98 ± 0.42	-2.90 ± 0.10	-2.15 ± 0.44
*p-value*	**0.002**		0.173		0.072		0.762		0.107	
**Verbal memory (List learning)**	-1.98 ± 0.14	-2.23 ± 0.13	-2.14 ± 0.12	-2.08 ± 0.15	-1.77 ± 0.18	-2.25 ± 0.11	-2.09 ± 0.09	-2.55 ± 0.38	-2.16 ± 0.09	-1.46 ± 0.40
*p-value*	0.197		0.743		**0.037**		0.241		0.098	
**Working memory (Digit sequencing)**	-1.68 ± 0.16	-2.18 ± 0.15	-1.86 ± 0.14	-2.11 ± 0.18	-1.75 ± 0.22	-2.03 ± 0.13	-1.95 ± 0.11	-2.02 ± 0.45	-1.99 ± 0.11	-1.40 ± 0.48
*p-value*	**0.032**		0.284		0.313		0.881		0.242	
**Motor speed (Token motor task)**	-2.13 ± 0.13	-2.54 ± 0.12	-2.27 ± 0.11	-2.52 ± 0.14	-2.13 ± 0.18	-2.45 ± 0.10	-2.37 ± 0.09	-2.25 ± 0.36	-2.37 ± 0.09	-2.21 ± 0.39
*p-value*	**0.027**		0.184		0.154		0.761		0.702	
**Verbal fluency (Semantic, alphabetical**)	-1.26 ± 0.12	-1.72 ± 0.11	-1.42 ± 0.11	-1.67 ± 0.14	-1.39 ± 0.17	-1.56 ± 0.10	-1.51 ± 0.09	-1.63 ± 0.35	-1.56 ± 0.08	-0.73 ± 0.36
*p-value*	**0.009**		0.175		0.409		0.734		**0.030**	
**Attention and speed of information processing (Symbol coding)**	-2.07 ± 0.15	-2.69 ± 0.13	-2.26 ± 0.13	-2.65 ± 0.17	-2.24 ± 0.21	-2.48 ± 0.12	-2.42 ± 0.11	-2.27 ± 0.43	-2.44 ± 0.10	-1.88 ± 0.45
*p-value*	**0.004**		0.083		0.351		0.740		0.234	
**Executive function (Tower of London)**	-1.57 ± 0.28	-2.56 ± 0.25	-2.02 ± 0.25	-2.28 ± 0.32	-1.50 ± 0.38	-2.36 ± 0.23	-2.07 ± 0.20	-2.77 ± 0.79	-2.17 ± 0.20	-1.15 ± 0.83
*p-value*	**0.014**		0.518		0.069		0.397		0.240	

Note: the association between the antipsychotics used and the BACS total score and subtests was adjusted for treatment duration, illness length, and chlorpromazine equivalent dose

Mood stabilizer: lithium carbonate and Antiepileptics: Antiepileptics: valproic acid, carbamazepine, pregabalin, topiramate, phenytoin

Benzodiazepines: lorazepam, clonazepam, alprazolam, diazepam, alprazolam, bromazepam

Anticholinergics: trihexyphenidyl

Tricyclic antidepressants: amitriptyline, clomipramine, imipramine

other antidepressants: venlafaxine, sertraline

### Associations of ADS, antipsychotic dose, and number of antipsychotics used with cognitive function

Total objective cognitions (BACS total score), verbal memory, and motor speed were inversely associated with ADS. Higher BACS total score (*r* = -0.18, *p* = 0.04), higher verbal memory (*r* = -0.21, *p* = 0.02), and higher motor speed (*r* = -0.19, *p* = 0.03) were significantly associated with lower ADS scores. Also, a significantly higher total PANSS score (*r* = 0.18, *p* = 0.04), positive PANSS (*r* = 0.18, *p* = 0.04), and general psychopathology (*r* = 0.19, *p* = 0.03) were significantly associated with higher ADS scores.

A significantly higher BACS total score (*r* = -0.33, *p* < 0.001), higher verbal memory (*r* = -0.26, *p* = 0.004), higher working memory (*r* = -0.20, *p* = 0.03), higher motor speed (*r* = -0.36, *p* < 0.001), and higher attention and speed of information processing (*r* = -0.27, *p* = 0.003) were associated with a lower chlorpromazine equivalent dose.

No significant association was found between the number of antipsychotics used and cognition (*p* > 0.05 for all) (Table 5).

**Table 5:** Correlation coefficient between antipsychotic and anticholinergic treatment and cognitive function

	**ADS total score**	**Chlorpromazine equivalent dose**	**Number of antipsychotics used**
	**Correlation coefficient**	**Correlation coefficient**	**Correlation coefficient**
**BACS (global score)**	-.182	-.332	-.028
*p-value*	**.046**	**< .0001**	.763
**Verbal memory (List learning)**	-.212	-.269	-.108
*p-value*	**.020**	**.004**	.239
**Working memory (Digit sequencing)**	-.036	-.203	.055
*p-value*	.699	**.031**	.549
**Motor speed (Token motor task)**	-.192	-.369	-.008
*p-value*	**.036**	**< .0001**	.934
**Verbal fluency (Semantic, alphabetical**)	-.121	-.160	-.027
*p-value*	.187	.090	.768
**Attention and speed of information processing (Symbol coding)**	-.113	-.275	-.064
*p-value*	.218	**.003**	.485
**Executive function (Tower of London)**	120	113	.076
*p-value*	-.128	-.183	.409
**Total PANSS scale**	.186	.109	.169
*p-value*	**.042**	.249	.065
**Positive PANSS scale**	.187	.073	.210
*p-value*	**.041**	.441	**.021**
**Negative PANSS scale**	.007	.012	.071
*p-value*	.940	.899	.439
**General psychopathology PANSS scale**	.190	.130	.120
*p-value*	**.038**	.171	.194
**Depression**	-.095	-.130	-.129
*p-value*	.300	.170	.160
**Autonomy**	-.002	.050	.129
*p-value*	.983	.597	.162

Anticholinergic drug scale (ADS)

**Fig. 1:**
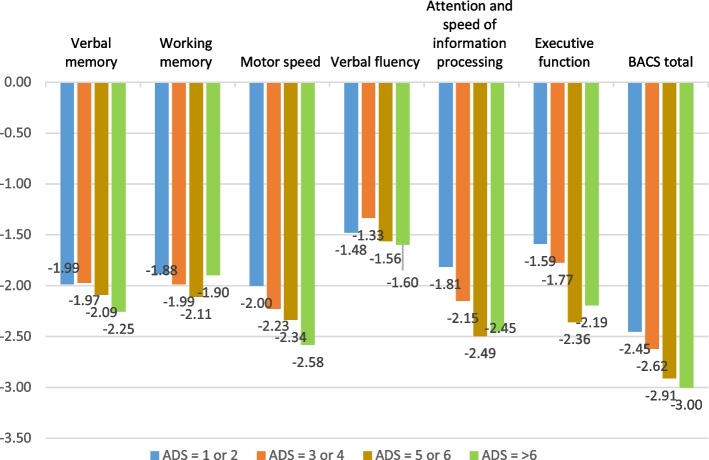
Association between the categories of Anticholinergic drug scale (ADS) and cognitive domains

In our sample, 62 participants (34.4%) had an ADS score over 6, 18.9% scored between 5 and 6, 12.2% scored between 3 and 4, and only 1.1% had an ADS score ranging from 1 to 2. As shown in Fig. 1, the higher the anticholinergic burden, the worse the cognitive performance. Motor speed was the domain most associated with ADS score severity, followed by attention and speed of information processing.

### Multivariable analysis

Multivariable linear regressions were conducted, taking the BACS total score and subtests as the dependent variables adjusted for gender, education level, age, duration of illness, total PANSS score, and depression.

The first linear regression taking the BACS total score as the dependent variable showed that higher ADS scores (Beta = -0.08; *p* = 0.028), higher chlorpromazine equivalent dose (Beta = -0.0003; *p* = 0.001), and taking mood stabilizers (Beta = -0.57; *p* = 0.004) were significantly associated with lower cognition.

In the other models taking the subtests of the BACS scale as the dependent variables, a higher chlorpromazine equivalent dose was inversely associated with all subtests except verbal fluency. However, a higher ADS total score was significantly associated with lower motor speed (Beta = -0.08; *p* = 0.011) and lower executive function (Beta = -0.14; *p* = 0.041). Taking mood stabilizers was significantly associated with lower working memory (Beta = -0.50; *p* = 0.018), verbal fluency (Beta = -0.43; *p* = 0.016), attention and speed of information processing (Beta = -0.65; *p* = 0.002), and executive function (Beta = -0.80; *p* = 0.030). A higher number of antipsychotics was related to higher executive function (Beta = 0.67; *p* = 0.013) (Table 6).

**Table 6:** Multivariable analysis

	**UB**	**SB**	**p-value**	**CI**	
				**Lower**	**Upper**
**Model 1: linear regression taking the BACS total score as the dependent variable**					
ADS	-.084	-.218	**.028**	-.159	-.009
Chlorpromazine equivalent dose	-.0003	-.307	**.001**	-.001	-.00001
Number of antipsychotics	.195	.150	.176	-.089	.480
Mood stabilizer medications (Yes vs. No*)	-.579	-.245	**.004**	-.967	-.192
Adjusted R2: .313					
**Model 2: linear regression taking the Verbal memory score as the dependent variable**					
ADS	-.048	-.143	.195	-.121	.025
Chlorpromazine equivalent dose	-.0001	-.207	**.041**	-.0003	-.00008
Number of antipsychotics	.042	.037	.765	-.235	.319
Mood stabilizer medications (Yes vs. No*)	-.182	-.088	.341	-.558	.195
Adjusted R2: .214					
**Model 3: linear regression taking the Working memory score as the dependent variable**					
ADS	-.043	-.112	.288	-.124	.037
Chlorpromazine equivalent dose	-.0002	-.225	**.021**	-.0004	-.00003
Number of antipsychotics	.293	.225	.060	-.013	.599
Mood stabilizer medications (Yes vs. No*)	-.504	-.213	**.018**	-.920	-.088
Adjusted R2: .170					
**Model 4: linear regression taking the Motor speed score as the dependent variable**					
ADS	-.084	-.256	**.011**	-.149	-.019
Chlorpromazine equivalent dose	-.0003	-.394	** < .001**	-.0005	-.0001
Number of antipsychotics	.105	.095	.397	-.141	.351
Mood stabilizer medications (Yes vs. No*)	-.306	-.152	.072	-.641	.028
Adjusted R2: .291					
**Model 5: linear regression taking the Verbal fluency score as the dependent variable**					
ADS	-.038	-.125	.264	-.106	.029
Chlorpromazine equivalent dose	-0.0008	-.106	.297	-.0002	.00008
Number of antipsychotics	.070	.067	.592	-.189	.329
Mood stabilizer medications (Yes vs. No*)	-.436	-.231	**.016**	-.788	-.083
Adjusted R2: .109					
**Model 6: linear regression taking the Attention and speed of information processing score as the dependent variable**					
ADS	-.047	-.124	.238	-.125	.031
Chlorpromazine equivalent dose	-.0002	-.219	**.024**	-.0004	-.00002
Number of antipsychotics	.111	.087	.462	-.186	.407
Mood stabilizer medications (Yes vs. No*)	-.652	-.283	**.002**	-1.056	-.248
Adjusted R2: 0.211					
**Model 7: linear regression taking the Executive function score as the dependent variable**					
ADS	-.145	-.216	**.041**	-.283	-.006
Chlorpromazine equivalent dose	-.0003	-.198	**.040**	-.001	-.00001
Number of antipsychotics	.672	.297	**.013**	.144	1.200
Mood stabilizer medications (Yes vs. No*)	-.804	-.196	**.030**	-1.523	-.086
Adjusted R2: .217					

The models were adjusted for gender, education level, age, illness length, total PANSS score, and depression

Mood stabilizer: lithium carbonate and Antiepileptics: valproic acid, carbamazepine, pregabalin, topiramate, phenytoin

*UB* Unstandardized beta, *SB* Standardized beta, *CI* Confidence interval

## Discussion

This study aimed to evaluate the association between neuropsychological medications, their anticholinergic burden and antipsychotic doses, and cognition in chronic patients with schizophrenia. The findings confirmed that anticholinergic and antipsychotic doses are inversely related to cognition, as people receiving higher doses of anticholinergics and antipsychotics performed worse on cognitive tests; it also showed that mood stabilizers had an independent effect. Specifically, a higher anticholinergic burden was associated with poorer verbal memory and motor speed tasks; however, this association seemed to be small. Nonetheless, the use of anticholinergic agents is a therapeutic concern, particularly in a population where cognitive impairment is prevalent, and it has been demonstrated that these agents influence functioning. Other studies among patients with schizophrenia have shown similar results, where poor cognition was associated with higher anticholinergic and antipsychotic doses [34, 44–46]. A recent systematic review that included 17 articles of different methodological designs found that medications with a higher anticholinergic load have a detrimental impact on neurocognitive function in patients with schizophrenia [23]. A meta-analysis of 34 studies found no association between antipsychotic dose and cognition [13]. Another meta-analysis that included two randomized clinical trials showed that reducing by 50% antipsychotic doses considerably improved neurocognitive function [47]. The discrepancy between some data from previous studies and our findings could be explained by the differences in follow-up time, setting, selected population, antipsychotic doses, and the lack of reported data on medication doses.

Our results showed that the cognitive domains associated with ADS scores were verbal memory and motor speed; however, in the regression analysis, after adjusting for confounders, the associated domains became motor speed and executive functions. Regarding the antipsychotic dose, verbal memory, working memory, motor speed, and attention domains were significantly associated with chlorpromazine doses; these domains, in addition to the executive function, remained significant after adjustment. Similar results were found in a study among patients with psychotic disorders, where the verbal memory subtest of the BACS was mainly related to anticholinergic burden [35]. Additionally, a worse token motor score of the BACS was associated with increasing doses of antipsychotic medications [35]. Another study revealed that ADS is solely associated with the memory/fluency factor [34]. In chronic outpatients schizophrenia, the serum anticholinergic activity was associated with verbal memory, working memory, and verbal learning [44]. There is strong evidence that a higher anticholinergic load decreases cognition, specifically verbal memory, attention and executive function and that reducing anticholinergic prescription dosages improves cognitive tasks [23]. These inconsistencies in findings might be due to the different measures of anticholinergic burden and the variability of the diagnostic tools used to test cognitive domains.

In this study, the average ADS score was 7.18, higher than the previously reported values of about 3.8 [33, 34]. In our data, the proportion of patients with an ADS score of at least 4 was 46.6%, with 34.4% having an ADS score ≥ 6. A possible explanation for the higher value is that patients with chronic psychotic disorders residing in a psychiatric institute receive recurrent daily doses of antipsychotics and are more likely to be prescribed multiple medications, which raises the risk and severity of the anticholinergic medication burden. In chronic patients with psychiatric conditions, polypharmacy is a common occurrence that results in the increased use of anticholinergics. These patients might also have a severe medical illness that could likely increase the ADS score by using additional medications with anticholinergic properties. Antipsychotics accounted for more than half of the anticholinergic load, with the rest coming from classic anticholinergics, antidepressants, mood stabilizers, and benzodiazepines.

Our results showed that the chlorpromazine equivalent dose affected cognition, whatever the type of antipsychotics (first and second-generation), after adjustment for illness length and treatment duration. Studies have demonstrated that high antipsychotic doses are associated with several cognitive deficits [17, 48–50] and a detrimental effect on learning, attention, and processing speed in healthy subjects compared to a placebo [51]. Kontis and colleagues found no differences in cognitive scores between patients with schizophrenia receiving excessive daily doses of chlorpromazine equivalents and patients receiving normal doses; however, their findings might have been confounded by their definition of the normal dose [52]. Also, patients were treated with two or more antipsychotics, which is a risk factor for excessive dosing, thus affecting cognitive function. The mean chlorpromazine dose in this study was over 1000 mg daily, indicating that patients are taking high doses because of more severe symptoms (disease) or poor medication compliance, which may be related to the prescription of high-dose antipsychotics. In addition, it could be that the methodological approach used to determine the dose equivalents for chlorpromazine equivalent and the inaccuracies in dose conversions for antipsychotics could introduce some bias in the calculation.

Our results also revealed that participants taking mood stabilizers had significantly lower cognition, independent of other effects. Mood stabilizers have already been associated with cognitive adverse effects [53, 54]. Mood stabilizers such as lithium and anticonvulsant drugs valproate and lamotrigine are used as an adjunct in schizophrenia to improve treatment effectiveness [55]. In our study, mood stabilizers (lithium and antiepileptic medications), although not very anticholinergic, and prescribed with antipsychotics, were significantly associated with worse cognitive deficits of any type (verbal memory, executive functions, etc.). This result probably reflects the presence of a resistant form of schizophrenia in patients taking these medications, thus, more severe cases of the disease with higher cognitive deficits. Of note, the prescription of lithium with antipsychotic drugs in schizophrenia has not shown to be effective [56]. Finally, many mood stabilizers have anticholinergic properties, and giving them to patients taking antipsychotics worsens their anticholinergic load with a significant risk of adverse cognitive effects. Each prescriber should consider this risk/benefit balance when prescribing these molecules, even in chronic patients, and should not hesitate to modify these regimens in the case of significant cognitive deterioration or less cognitive tolerance, particularly in older patients. When undergoing neuropsychological testing, patients with schizophrenia are frequently given a combination of antipsychotics, mood stabilizers, neuroleptics, anticonvulsants, benzodiazepines, and antidepressants. The neuropsychological assessment might be confounded by multiple-medication regimens and may or may not alter cognitive test results.

The number of antipsychotics may increase cognitive performance to some extent, with the degree of benefit varying depending on the cognitive domain and anticholinergic burden. Treatment with antipsychotics might be associated with a modest positive impact on cognitive functioning [57]. However, the association between the number of antipsychotics and cognitive improvement might be complicated by the presence of confounding variables [57]. Also, long-term treatment, higher doses of antipsychotics, and polypharmacy might potentially harm the brain structure and impair cognitive performance [58–60]. This study could not establish a causal association between the drug type (FGA/SGA) and cognitive performance. More extensive longitudinal studies are necessary to clarify the cause-effect relationship in patients with schizophrenia.

### Clinical implication

Even moderate doses of antipsychotics might impair cognitive performance; hence, a well-designed psychopharmacological medication plan is essential. High doses of antipsychotics might help with symptoms, including delusions and hallucinations. Nonetheless, relapse-prevention medications should be chosen cautiously, considering cognitive and functional outcomes. Although these effects may differ from patient to patient, the rationale for prescribing medicines should be kept in mind. When the antipsychotic dose is kept in the normal-low range, and polypharmacy is avoided, as indicated in the EUFEST Study, a slightly favorable effect on cognitive performance can be identified [61]. As a result, the pharmacological treatment plan should only comprise the minimum dose of antipsychotics and avoid the add-on of anticholinergics. Also, it would be of interest if clinicians and other health professionals evaluated the long-term impact of anticholinergic medications on cognition.

### Limitations

This study has several limitations. First, its cross-sectional design makes it hard to evaluate the long-term effects of anticholinergic burden on cognitive performance in patients with schizophrenia or evaluate the causal relation between anticholinergic burden and cognition. Second, the results could not be generalized to all individuals with schizophrenia due to the small sample size and because participants were selected from a single site (a tertiary care hospital). Third, participants were hospitalized chronically with more severe illness and poorer functioning as compared to acute patients, which might have introduced a selection bias in interpreting the results. Our findings may not apply to clinical high-risk, prodromal, or early-stage disease populations, and the link between ADS scores and cognitive performance may change during acute phases or immediately after remission. Also, information was self-reported by the participants, which could have produced information bias since exact details could not be provided during the face-to-face interview. In addition, the anticholinergic drug scale (ADS) cannot calculate systemic drug exposure, brain delivery, distribution of medications, and drug interactions that can often affect the overall anticholinergic activity. Furthermore, it cannot be utilized to conclude which medications should be stopped to lower the anticholinergic burden. The gold standard for assessing anticholinergic activity is to measure serum anticholinergic rate; however, this measure may only reflect a transitional cholinergic condition outside the brain, which provides an intuitive clinical capability but lacks a direct in vivo assessment of the central effects of anticholinergic medications. The association found between cognition and medications might be due to the disease per se since no control group nor a baseline value was considered for comparison. The medical conditions of participants were not assessed, which might influence the anticholinergic charge. The use of propensity scores in future studies is suggested. Residual confounding bias could have occurred since not all factors related to ADS and cognition were tested.

## Conclusion

This study confirms that the cognitive functions of chronic patients with schizophrenia may be affected by medications and their anticholinergic burden. Different cognitive domains were inversely associated with the anticholinergic burden in these patients. Well-designed large-sample prospective studies and randomized clinical trials are necessary to investigate the effect of anticholinergic medications on cognition in patients with schizophrenia. These studies would examine anticholinergic drug exposure over time on larger samples and a more extended monitoring period to minimize as many systemic biases as feasible and draw more general results. More neurological research is also needed to clarify the role of cholinergic neurotransmission and neurochemical mechanisms in the cognitive impairment of patients with schizophrenia.

## Supplementary Information

**Additional file 1:** **Table S1.** Association between the doses of medication and the cognitive function among patients with schizophrenia.

## Data Availability

Data can be made available under reasonable request form the corresponding author.

## Abbreviations

AP: Antipsychotic
ADS: Anticholinergic drug scale
ACB: Anticholinergic cognitive burden
BACS: Brief assessment of cognition in schizophrenia
CATIE: Clinical antipsychotic trials of intervention effectiveness
CI: Confidence interval
DSM-5: Diagnostic and statistical manual of mental disorders, fifth edition
FGA: First generation of antipsychotics
PANSS: Positive and negative syndrome scale
SGA: Second generation of antipsychotics
TCA: Tricyclic antidepressants
SSRI: Selective serotonin reuptake inhibitors
SPSS: Statistical package for social sciences
SB: Standardized beta
SD: Standard deviation
UB: Unstandardized beta

## References

[1] Heinrichs RW, Zakzanis KK (1998). Neurocognitive deficit in schizophrenia: a quantitative review of the evidence. Neuropsychology.

[2] Keefe RS, Eesley CE, Poe MP (2005). Defining a cognitive function decrement in schizophrenia. Biol Psychiat.

[3] Bora E, Murray RM (2014). Meta-analysis of cognitive deficits in ultra-high risk to psychosis and first-episode psychosis: do the cognitive deficits progress over, or after, the onset of psychosis?. Schizophr Bull.

[4] Rajji TK, Miranda D, Mulsant BH (2014). Cognition, function, and disability in patients with schizophrenia: a review of longitudinal studies. The Canadian Journal of Psychiatry.

[5] Dickinson D, Ragland JD, Gold JM, Gur RC (2008). General and specific cognitive deficits in schizophrenia: Goliath defeats David?. Biol Psychiat.

[6] Nuechterlein KH, Barch DM, Gold JM, Goldberg TE, Green MF, Heaton RK (2004). Identification of separable cognitive factors in schizophrenia. Schizophr Res.

[7] Haddad C, Salameh P, Sacre H, Clément J-P, Calvet B (2021). General description of cognitive deficits in schizophrenia and assessment tools in Lebanon: A scoping review. Schizophrenia Research: Cognition.

[8] Green MF (1996). What are the functional consequences of neurocognitive deficits in schizophrenia?. The Am J Psychiatry.

[9] Green MF, Kern RS, Braff DL, Mintz J (2000). Neurocognitive deficits and functional outcome in schizophrenia: are we measuring the “right stuff”?. Schizophr Bull.

[10] Hofer A, Baumgartner S, Bodner T (2005). Patient outcomes in schizophrenia II: the impact of cognition. Eur Psychiatry.

[11] Désaméricq G, Schurhoff F, Meary A (2014). Long-term neurocognitive effects of antipsychotics in schizophrenia: a network meta-analysis. Eur J Clin Pharmacol.

[12] Woodward ND, Purdon SE, Meltzer HY, Zald DH (2005). A meta-analysis of neuropsychological change to clozapine, olanzapine, quetiapine, and risperidone in schizophrenia. Int J Neuropsychopharmacol.

[13] Mishara AL, Goldberg TE (2004). A meta-analysis and critical review of the effects of conventional neuroleptic treatment on cognition in schizophrenia: opening a closed book. Biol Psychiat.

[14] MacKenzie NE, Kowalchuk C, Agarwal SM (2018). Antipsychotics, metabolic adverse effects, and cognitive function in schizophrenia. Front Psych.

[15] Keefe RS, Bilder RM, Davis SM (2007). Neurocognitive effects of antipsychotic medications in patients with chronic schizophrenia in the CATIE Trial. Arch Gen Psychiatry.

[16] Woodward ND, Purdon SE, Meltzer HY, Zald DH (2007). A meta-analysis of cognitive change with haloperidol in clinical trials of atypical antipsychotics: dose effects and comparison to practice effects. Schizophr Res.

[17] Elie D, Poirier M, Chianetta J, Durand M, Grégoire C, Grignon S (2010). Cognitive effects of antipsychotic dosage and polypharmacy: a study with the BACS in patients with schizophrenia and schizoaffective disorder. J Psychopharmacol.

[18] Torniainen M, Suvisaari J, Partonen T (2012). Cognitive impairments in schizophrenia and schizoaffective disorder: relationship with clinical characteristics. J Nerv Ment Dis.

[19] Takeuchi H, Suzuki T, Remington G (2013). Effects of risperidone and olanzapine dose reduction on cognitive function in stable patients with schizophrenia: an open-label, randomized, controlled, pilot study. Schizophr Bull.

[20] Graff-Guerrero A, Rajji TK, Mulsant BH (2015). Evaluation of antipsychotic dose reduction in late-life schizophrenia: a prospective dopamine D2/3 receptor occupancy study. JAMA Psychiat.

[21] Irani F, Kalkstein S, Moberg EA, Moberg PJ (2011). Neuropsychological performance in older patients with schizophrenia: a meta-analysis of cross-sectional and longitudinal studies. Schizophr Bull.

[22] Campbell N, Boustani M, Limbil T (2009). The cognitive impact of anticholinergics: a clinical review. Clin Interv Aging.

[23] Georgiou R, Lamnisos D, Giannakou K. Anticholinergic burden and cognitive performance in patients with schizophrenia: a systematic literature review. Frontiers in psychiatry. 2021;12:779607. 10.3389/fpsyt.2021.779607 PMC8748260 35027893

[24] Chakos M, Patel J, Rosenheck R (2011). Concomitant psychotropic medication use during treatment of schizophrenia patients: longitudinal results from the CATIE study. Clin Schizophr Relat Psychoses.

[25] Briet J, Javelot H, Heitzmann E (2017). The anticholinergic impregnation scale: Towards the elaboration of a scale adapted to prescriptions in French psychiatric settings. Therapies.

[26] Ogino S, Miyamoto S, Miyake N, Yamaguchi N (2014). Benefits and limits of anticholinergic use in schizophrenia: focusing on its effect on cognitive function. Psychiatry Clin Neurosci.

[27] Biringer E, Rongve A, Lund A (2009). A review of modern antidepressants' effects on neurocognitive function. Current Psychiatry Reviews.

[28] Carnahan RM, Lund BC, Perry PJ, Pollock BG, Culp KR (2006). The anticholinergic drug scale as a measure of drug-related anticholinergic burden: associations with serum anticholinergic activity. J Clin Pharmacol.

[29] Albert M. Anticholinergic discontinuation for antipsychotic-induced extra-pyramidal symptoms. 2017.

[30] Ancelin ML, Artero S, Portet F, Dupuy A-M, Touchon J, Ritchie K (2006). Non-degenerative mild cognitive impairment in elderly people and use of anticholinergic drugs: longitudinal cohort study. BMJ.

[31] Risacher SL, McDonald BC, Tallman EF (2016). Association between anticholinergic medication use and cognition, brain metabolism, and brain atrophy in cognitively normal older adults. JAMA Neurol.

[32] Ehrt U, Broich K, Larsen JP, Ballard C, Aarsland D (2010). Use of drugs with anticholinergic effect and impact on cognition in Parkinson's disease: a cohort study. J Neurol Neurosurg Psychiatry.

[33] Joshi YB, Thomas ML, Braff DL, et al. Anticholinergic Medication Burden–Associated Cognitive Impairment in Schizophrenia. American Journal of Psychiatry. 2021;178(9):838-47. 10.1176/appi.ajp.2020.20081212 PMC8440496 33985348

[34] San Ang M, Rashid NAA, Lam M (2017). The impact of medication anticholinergic burden on cognitive performance in people with schizophrenia. J Clin Psychopharmacol.

[35] Eum S, Hill SK, Rubin LH (2017). Cognitive burden of anticholinergic medications in psychotic disorders. Schizophr Res.

[36] Tsoutsoulas C, Mulsant BH, Kumar S (2017). Anticholinergic burden and cognition in older patients with schizophrenia. The J Clin Psychiatry.

[37] Haddad C, Salameh P, Hallit S (2021). Cross-cultural adaptation and validation of the Arabic version of the BACS scale (the brief assessment of cognition in schizophrenia) among chronic schizophrenic inpatients. BMC Psychiatry.

[38] Carnahan RM, Lund BC, Perry PJ, Culp KR, Pollock BG (2002). The relationship of an anticholinergic rating scale with serum anticholinergic activity in elderly nursing home residents. Psychopharmacol Bull.

[39] Andreasen NC, Pressler M, Nopoulos P, Miller D, Ho B-C (2010). Antipsychotic dose equivalents and dose-years: a standardized method for comparing exposure to different drugs. Biol Psychiat.

[40] ClinCalc. Equivalent Benzodiazepine Calculator. Available at: https://clincalc.com/Benzodiazepine/. [Last Accessed 21 December, 2022]. 2022.

[41] Keefe RS, Goldberg TE, Harvey PD, Gold JM, Poe MP, Coughenour L (2004). The Brief Assessment of Cognition in Schizophrenia: reliability, sensitivity, and comparison with a standard neurocognitive battery. Schizophr Res.

[42] Hallit S, Obeid S, Haddad C, Kazour F, Kazour G. Validation of the Arabic Version of the PANSS scale among Lebanese schizophrenic patients. Journal of Psychopathology. 2017.

[43] Kay SR, Fiszbein A, Opler LA (1987). The positive and negative syndrome scale (PANSS) for schizophrenia. Schizophr Bull.

[44] Vinogradov S, Fisher M, Warm H, Holland C, Kirshner MA, Pollock BG (2009). The cognitive cost of anticholinergic burden: decreased response to cognitive training in schizophrenia. Am J Psychiatry.

[45] Minzenberg MJ, Poole JH, Benton C, Vinogradov S (2004). Association of anticholinergic load with impairment of complex attention and memory in schizophrenia. Am J Psychiatry.

[46] Rehse M, Bartolovic M, Baum K, Richter D, Weisbrod M, Roesch-Ely D (2016). Influence of antipsychotic and anticholinergic loads on cognitive functions in patients with schizophrenia. Schizophr Res Treatment.

[47] Tani H, Takasu S, Uchida H, Suzuki T, Mimura M, Takeuchi H (2020). Factors associated with successful antipsychotic dose reduction in schizophrenia: a systematic review of prospective clinical trials and meta-analysis of randomized controlled trials. Neuropsychopharmacology.

[48] Hori H, Noguchi H, Hashimoto R (2006). Antipsychotic medication and cognitive function in schizophrenia. Schizophr Res.

[49] Moritz S, WoodwardKrausz TM, Naber D, Group PS (2002). Relationship between neuroleptic dosage and subjective cognitive dysfunction in schizophrenic patients treated with either conventional or atypical neuroleptic medication. Int Clin Psychopharmacol.

[50] Hori H, Yoshimura R, Katsuki A (2012). Several prescription patterns of antipsychotic drugs influence cognitive functions in Japanese chronic schizophrenia patients. Int J Psychiatry Clin Pract.

[51] Veselinović T, Schorn H, Vernaleken IB (2013). Effects of antipsychotic treatment on cognition in healthy subjects. J Psychopharmacol.

[52] Kontis D, Theochari E, Kleisas S (2010). Doubtful association of antipsychotic polypharmacy and high dosage with cognition in chronic schizophrenia. Prog Neuropsychopharmacol Biol Psychiatry.

[53] Honig A, Arts B, Ponds R, Riedel W. Lithium induced cognitive side-effects in bipolar disorder: a qualitative analysis and implications for daily practice. International clinical psychopharmacology. 1999. 10435769

[54] Pachet AK, Wisniewski AM (2003). The effects of lithium on cognition: an updated review. Psychopharmacology.

[55] Citrome L (2009). Adjunctive lithium and anticonvulsants for the treatment of schizophrenia: what is the evidence?. Expert Rev Neurother.

[56] Leucht S, Helfer B, Dold M, Kissling W, McGrath JJ. Lithium for schizophrenia. Cochrane Database of Systematic Reviews. 2015;(10):CD003834. 10.1002/14651858.CD003834. 10.1002/14651858.CD003834.pub3 PMC6984626 26509923

[57] MacKenzie NE, Kowalchuk C, Agarwal SM (2018). Antipsychotics, metabolic adverse effects, and cognitive function in schizophrenia. Front Psychiatry.

[58] Veijola J, Guo JY, Moilanen JS (2014). Longitudinal changes in total brain volume in schizophrenia: relation to symptom severity, cognition and antipsychotic medication. PLoS ONE.

[59] Andreasen NC, Liu D, Ziebell S, Vora A, Ho B-C (2013). Relapse duration, treatment intensity, and brain tissue loss in schizophrenia: a prospective longitudinal MRI study. Am J Psychiatry.

[60] Fusar-Poli P, Smieskova R, Kempton M, Ho B, Andreasen N, Borgwardt S (2013). Progressive brain changes in schizophrenia related to antipsychotic treatment? A meta-analysis of longitudinal MRI studies. Neurosci Biobehav Rev.

[61] Davidson M, Galderisi S, Weiser M (2009). Cognitive effects of antipsychotic drugs in first-episode schizophrenia and schizophreniform disorder: a randomized, open-label clinical trial (EUFEST). Am J Psychiatry.

