# Supplementary material for: Effects of antipsychotic and anticholinergic medications on cognition in chronic patients with schizophrenia
Source: BMC Psychiatry. 2023 Jan 24;23:61. doi: 10.1186/s12888-023-04552-y (PMC9872384; doi:10.1186/s12888-023-04552-y)
Supplement: Supplementary file 1 — Additional file 1: Table S1. Association between the doses of medication and the cognitive function among patients with schizophrenia. [file 12888_2023_4552_MOESM1_ESM.docx]

| Table S1: Association between the doses of medication and the cognitive function among patients with schizophrenia | | | | | | | | |
| --- | --- | --- | --- | --- | --- | --- | --- | --- |
| Doses of medication | | BACS total | Verbal memory | Working memory | Motor speed | Verbal fluency | Attention and speed of information processing | Executive function |
| Lithium carbonate | Pearson Correlation | .167 | .148 | .183 | .135 | .178 | .054 | .210 |
|  | Sig. (2-tailed) | .536 | .585 | .498 | .617 | .510 | .842 | .435 |
|  | N | 16 | 16 | 16 | 16 | 16 | 16 | 16 |
| Valproic acid | Pearson Correlation | -0.002 | 0.040 | 0.045 | 0.018 | 0.144 | -0.073 | -0.193 |
|  | Sig. (2-tailed) | 0.986 | 0.780 | 0.757 | 0.902 | 0.319 | 0.613 | 0.179 |
|  | N | 50 | 50 | 50 | 50 | 50 | 50 | 50 |
| Carbamazepine | Pearson Correlation | -0.287 | -0.480 | -0.108 | 0.161 | -0.530 | -0.190 | 0.000 |
|  | Sig. (2-tailed) | 0.454 | 0.191 | 0.783 | 0.679 | 0.142 | 0.624 | 1.000 |
|  | N | 9 | 9 | 9 | 9 | 9 | 9 | 9 |
| Pregabalin | Pearson Correlation | .^b^ | .^b^ | .^b^ | .^b^ | .^b^ | .^b^ | .^b^ |
|  | Sig. (2-tailed) |  |  |  |  |  |  |  |
|  | N | 1 | 1 | 1 | 1 | 1 | 1 | 1 |
| Topiramate | Pearson Correlation | .^b^ | .^b^ | .^b^ | .^b^ | .^b^ | .^b^ | .^b^ |
|  | Sig. (2-tailed) |  |  |  |  |  |  |  |
|  | N | 1 | 1 | 1 | 1 | 1 | 1 | 1 |
| Phenytoin | Pearson Correlation | .^b^ | .^b^ | .^b^ | .^b^ | .^b^ | .^b^ | .^b^ |
|  | Sig. (2-tailed) |  |  |  |  |  |  |  |
|  | N | 1 | 1 | 1 | 1 | 1 | 1 | 1 |
| Benzodiazepine equivalent dose | Pearson Correlation | 0.095 | 0.153 | 0.170 | 0.024 | 0.092 | 0.006 | 0.111 |
|  | Sig. (2-tailed) | 0.551 | 0.333 | 0.281 | 0.880 | 0.562 | 0.970 | 0.486 |
|  | N | 42 | 42 | 42 | 42 | 42 | 42 | 42 |
| Trihexyphenidyl | Pearson Correlation | .167 | .148 | .183 | .135 | .178 | .054 | .210 |
|  | Sig. (2-tailed) | .536 | .585 | .498 | .617 | .510 | .842 | .435 |
|  | N | 16 | 16 | 16 | 16 | 16 | 16 | 16 |
| Amitriptyline | Pearson Correlation | -0.914 | -0.629 | -0.996 | -0.500 | -0.781 | **-1.000^**^** | -0.997 |
|  | Sig. (2-tailed) | 0.266 | 0.567 | 0.058 | 0.667 | 0.429 | **0.000** | 0.052 |
|  | N | 3 | 3 | 3 | 3 | 3 | 3 | 3 |
| Clomipramine | Pearson Correlation | -0.325 | -0.240 | -0.326 | -0.198 | -0.255 | -0.398 | -0.524 |
|  | Sig. (2-tailed) | 0.593 | 0.697 | 0.592 | 0.749 | 0.679 | 0.507 | 0.365 |
|  | N | 5 | 5 | 5 | 5 | 5 | 5 | 5 |
| Imipramine | Pearson Correlation | .^b^ | .^b^ | .^b^ | .^b^ | .^b^ | .^b^ | .^b^ |
|  | Sig. (2-tailed) |  |  |  |  |  |  |  |
|  | N | 2 | 2 | 2 | 2 | 2 | 2 | 2 |
| Venlafaxine | Pearson Correlation | .^b^ | .^b^ | .^b^ | .^b^ | .^b^ | .^b^ | .^b^ |
|  | Sig. (2-tailed) |  |  |  |  |  |  |  |
|  | N | 1 | 1 | 1 | 1 | 1 | 1 | 1 |
| Sertraline | Pearson Correlation | .266 | .281 | .618 | -.181 | .404 | .163 | .538 |
|  | Sig. (2-tailed) | .665 | .647 | .267 | .771 | .501 | .794 | .350 |
|  | N | 5 | 5 | 5 | 5 | 5 | 5 | 5 |
